# Surgical Excision of a Hemophilic Pseudotumor Causing Vascular Impingement in a Patient with Severe Hemophilia A: A Case Report

**DOI:** 10.1055/s-0044-1790594

**Published:** 2024-12-27

**Authors:** Juan Manuel Pradilla, Abelardo Tinoco, Luis Miguel Cely, Martha Paola Buitrago

**Affiliations:** 1 Departamento de Cirurgia Ortopédica, Fundación Cardioinfantil, Instituto de Cardiología, Bogotá, Colômbia

**Keywords:** hematoma, hemophilia A, hemorrhage, musculoskeletal system

## Abstract

Patients with hemophilia disease have a high risk of hemorrhage. Most hemorrhages can occur in the musculoskeletal system, presenting as hematomas, or, in rare occasions, as hemophilic pseudotumors, an uncommon pathology that are often misdiagnosed as musculoskeletal tumors because of their clinical behavior and characteristics on diagnostic imaging. Despite many treatment options, surgical excision is the treatment of choice. This article describes a case of hemophilia A in a patient who suffered from progressive swelling of the right thigh for 12 months. Diagnostic imaging suggested a hemophilia pseudotumor with vascular bundle impingement. Surgical excision was successful.

## Introduction

Haemophilia is a bleeding disorder that impairs the patient's ability to make blood clots and is caused by low levels of clotting factors VIII (hemophilia A) and IX (hemophilia B). Hemophilia can be classified as mild, moderate or severe hemophilia according to the number of factors VIII and IX produced. 1

Approximately 80% of patients with hemophilia suffer from a hemorrhage, mainly in the musculoskeletal system. When muscle hematomas appear, they can cause serious complications such as acute compartment syndrome, peripheral nerve compression and hemophilic pseudotumor, which is an uncommon complication with an incidence of 2%. 1 2 3

Fernandez de Valderrama and Matthews 4 (1965), note that a hemophilic pseudotumor is “progressive cystic swelling that involves the muscles, caused by recurrent hemorrhage and an accompaniment to radiographic evidence of bone involvement.” However, a hemophilic pseudotumor is considered to be an encapsulated hemorrhagic fluid collection caused by recurrent intramuscular, intraosseous or subperiosteal hemorrhages. They often occur in nonmusculoskeletal systems such as the lung, intra-abdominal organs, and subcutaneous tissue. 1 5 6

They have been divided into several classifications: Type I (occurs in soft tissue), Type II (in subperiosteal region) and Type III (in bone tissue). Gilbert, 7 classified them as either proximal or distal in terms of their location on the small bones of young patients. Fernandez de Valderrama and Matthews 4 described a distinctive type of hemophilic pseudotumor that affects the muscle and has no effect on the adjacent bone.

By 2022, Ziquian et al. 6 proposed an extensive classification of extremity and pelvic hemophilic pseudotumors, the PUMCH classification, according to the site of the pseudotumor on the extremity and the presence or absence of bone destruction.

The objective of this study is to report the presence of a soft tissue hemophilic pseudotumor in the thigh causing vascular bundle compression. Surgical excision and factor VII replacement therapy were performed.

## Case Report

The Case Report had Institutional Review Board IRB approval from our institution under number 010–2023. The patient consented to the publication of this article, authorizing the publication of clinical information, including physical and diagnostic images. The patient's name nor personal data will be published.

A 57-year-old man who suffered from progressive pain for 12 months and showed an increase in the circumferential diameter of the middle and proximal thirds of the thigh was admitted to the Emergency Department. He had motion and walking limitations and reported no prior trauma.

Examination of his medical history showed a diagnosis of type A hemophilia that was treated with Emicizumab (factor VIII) 90 mg weekly.

**Fig. 1:**
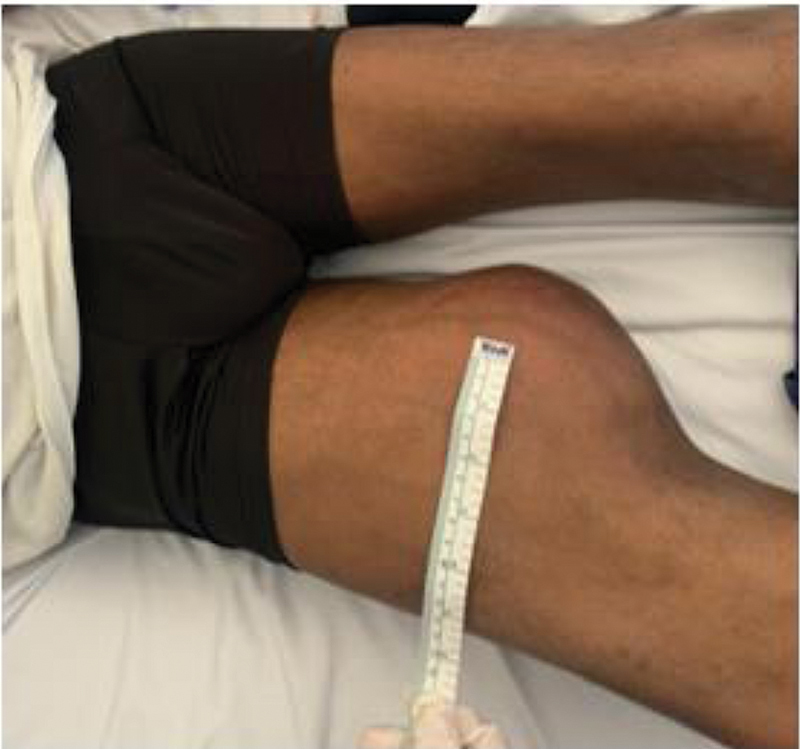
Physical examination showing that the circumference of the right thigh was larger than that of the contralateral limb. **Source:** *Authors archive.*

Physical examination showed swelling and that the circumference of the right thigh was 56 cm (22 cm wider than the left thigh). Pain and induration was observed upon palpation; peripheral, popliteal, dorsal pedis and posterior tibial pulses were present. No sensory or motor disturbances were found ( Fig. 1 ).

Upon admission, the patient had slightly decreased hemoglobin (HB) levels and platelet levels of 184,000 cells/u, which remained within normal ranges throughout hospitalization.

## Radiologic Images

The patient had a previous soft tissue ultrasound that showed three predominantly liquid collections in the middle third and inner thigh that extended toward the muscular planes. The largest of them measured 142*120*132 mm and had a volume of 1000 cc. The other two adjacent collections had volumes of 127 cc and 90 cc, respectively.

**Fig. 2:**
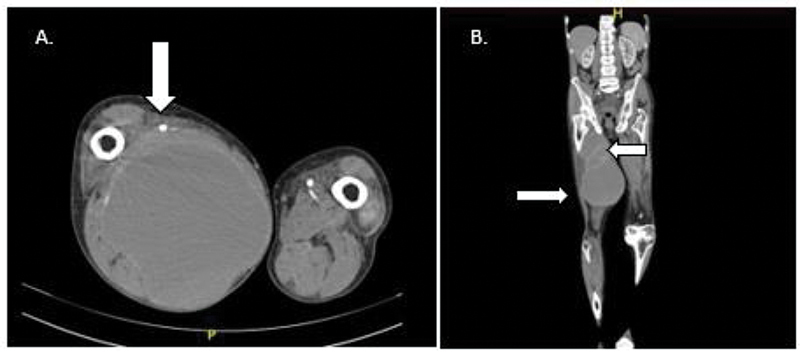
loculated collection at the muscular plane in the posteromedial region of the right thigh. The difference between the circumferences of the right and contralateral thighs is obvious. ( **A** ) CT angiography in an axial view showing vascular bundle impingement. ( **B** ) CT angiography in a coronal view. Capsular tissue surrounding the lesion can be seen. **Source** : Authors archive.

**Fig. 3:**
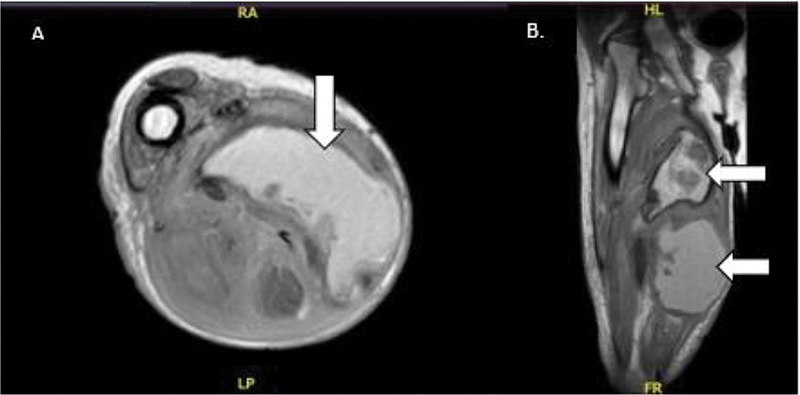
( **A** ) MRI scan T1, axial cut showing the presence of a collection measuring 103*57*182 mm in diameter in the medial compartment of the right thigh. ( **B** ) MRI scan T1, coronal cut showing multiple septate collections (indicated by the white arrow) extending to the subcutaneous cellular tissue. **Source:** Authors archive.

To evaluate vascular integrity, CT angiography was performed. It showed a loculated collection measuring 86*86*228 mm with an estimated volume of 884 cc at the muscular plane in the posteromedial region of the right thigh with characteristics suggesting a hematic component displacing anteriorly the vascular bundle of the extremity ( Figs. 2 and 3 ).

## Treatment

The patient underwent surgical excision. He received a first dose of 90 mcg/kg of recombinant factor VIIa (rFVIIa) (NovoSeven) intravenously (IV) during anesthetic induction and then 50 mcg/kg for 21 days after the intervention. A direct lateral retrovastus approach was performed, and intraoperative findings showed a well-defined, encapsulated hemorrhagic mass making contact with and displacing the vascular bundle of the extremity. Capsular tissue and 2000 cc of hematic content were excised. Percutaneous drainage was maintained for three days. A second surgical procedure was performed due to an increase in diameter, and 400 cc of hematic content was removed. The patient continued systemic therapy for 21 days and was discharged from the hospital without complications.

Following up with the patient after being discharged from the hospital, had its own challenges. Due to COVID-19 isolation laws, face-to-face control was unfeasible. Thus, two telephone follow-ups were made. The first control call, which took place three months after hospital discharge, the patient refers adequate clinical evolution, denying new bleeding episodes. Twelve months later, during the second control call, he referred a new bleeding episode that require recombinant factor VIIa application.

## Discussion

The pathophysiological basis of hemophilic pseudotumors involves recurrent bleeding causing encapsulated hematomas and a tumor-like appearance. They can cause erosion of the adjacent bony structures. 2 3 In most cases, there is a history of trauma or repeated bleeding due to factor VIII deficiency.

Hemophilic pseudotumors clinically present as a painless, expansive mass infiltrating most of the involved extremity. They can be found in the axial skeleton or in non-osseous tissues such as the lungs or intrabdominal organs. When hemophilic pseudotumors present in the appendicular skeleton, they can be associated with impaired joint motion and vascular or neurological deficits in rare cases.

There are few classification systems, such as the Gilbert Classification (according to localization) or the PUMCH classification, which is a classification system that considers localization, extension, and bone compromise.

Treatment includes observation and percutaneous drainage, but most hemophilic pseudotumors do not respond to conservative treatment. Surgical excision and systemic factor replacement therapy are the treatments of choice in most cases.

An uncontrollable, large or progressive hemophilic pseudotumor that is untreated can affect the integrity of the extremity, including vascular compression, neurological deficits or bone erosion/pathological fractures.

The relatively recent development of rFVIIa has allowed patients with high-titer inhibitors of factors VIII and IX to undergo surgical procedures safely. rFVIIa is a reliable hemostatic agent that does not have any thromboembolic side effects. Nevertheless, recurrent bleeding can occur despite of appropriate treatment. 1 2 8
