# Supplementary material for: Surgical Excision of a Hemophilic Pseudotumor Causing Vascular Impingement in a Patient with Severe Hemophilia A: A Case Report
Source: Rev Bras Ortop (Sao Paulo). 2024 Dec 27;59(Suppl 2):e216–9. doi: 10.1055/s-0044-1790594 (PMC11679610; doi:10.1055/s-0044-1790594)
Supplement: Related texts

# Research article: Excisão cirúrgica de um pseudotumor hemofílico causando impacto vascular em um paciente com hemofilia A grave: Relato de caso

**Keywords:** hematoma, hemofilia A, hemorragia, sistema musculoesquelético

## Abstract

Pacientes com doença hemofílica apresentam alto risco de hemorragia. A maioria das hemorragias pode ocorrer no sistema musculoesquelético, apresentando-se como hematomas ou, raramente, como pseudotumores hemofílicos, uma doença incomum e muitas vezes diagnosticada de forma errônea como tumores musculoesqueléticos devido ao seu comportamento clínico e a características em exames de diagnóstico por imagem. Apesar das muitas opções terapêuticas, a excisão cirúrgica é o tratamento de escolha. Este artigo descreve um caso de um paciente com hemofilia A que apresentou aumento de volume progressivo da coxa direita por 12 meses. Os exames de imagem sugeriram um pseudotumor hemofílico com pinçamento do feixe vascular. A excisão cirúrgica foi bem-sucedida.

## Introdução

A hemofilia é um distúrbio hemorrágico que prejudica a capacidade de formação de coágulos sanguíneos e é causada por baixos níveis de fatores de coagulação VIII (hemofilia A) e IX (hemofilia B). A hemofilia pode ser classificada como hemofilia branda, moderada ou grave de acordo com a quantidade de fatores VIII e IX produzidos. 1

Aproximadamente 80% dos pacientes com hemofilia sofrem de hemorragia, principalmente no sistema musculoesquelético. O aparecimento de hematomas musculares pode causar complicações graves, como síndrome compartimental aguda, compressão de nervo periférico e pseudotumor hemofílico, uma complicação incomum com incidência de 2%. 1 2 3

Fernandez de Valderrama e Matthews 4 (1965) notaram que um pseudotumor hemofílico é “aumento de volume cístico progressivo que acomete os músculos, é causado por hemorragia recorrente e requer acompanhamento de evidências radiográficas de envolvimento ósseo”. No entanto, um pseudotumor hemofílico é considerado uma coleção de fluido hemorrágico encapsulado decorrente de hemorragias intramusculares, intraósseas ou subperiósteas recorrentes. De modo geral, estes tumores ocorrem em sistemas não musculoesqueléticos, como o pulmão, os órgãos intra-abdominais e o tecido subcutâneo. 1 5 6

Há diversas classificações para os tumores hemofílicos: tipo I (que ocorre em tecidos moles), tipo II (na região subperióstea) e tipo III (no tecido ósseo). Gilbert ^7^ os classificou como proximais ou distais em termos de sua localização nos ossos pequenos de pacientes jovens. Fernandez de Valderrama e Matthews ^4^ descreveram um tipo distinto de pseudotumor hemofílico com acometimento muscular e sem efeitos no osso adjacente.

Em 2022, Ziquian et al. 6 propuseram uma classificação abrangente de pseudotumores hemofílicos pélvicos e de extremidades, a classificação do Peking Union Medical College Hospital (PUMCH), de acordo com a localização da lesão no membro e a presença ou ausência de destruição óssea.

O objetivo deste estudo foi relatar a presença de um pseudotumor hemofílico de partes moles na coxa causando compressão do feixe vascular. O tratamento consistiu em excisão cirúrgica e terapia de reposição de fator VII.

## Relato de Caso

O Relato de Caso teve aprovação do Comitê de Ética de nossa instituição sob o número 010–2023. O paciente consentiu com a publicação deste artigo, autorizando a divulgação de informações clínicas, incluindo imagens físicas e diagnósticas. O nome do paciente nem seus dados pessoais serão publicados.

Um homem de 57 anos que sofria de dor progressiva há 12 meses e apresentava aumento do diâmetro circunferencial dos terços médio e proximal da coxa foi admitido no Pronto-Socorro. O paciente tinha limitações de movimento e deambulação e relatou ausência de trauma prévio.

Seu prontuário médico revelou o diagnóstico de hemofilia tipo A tratada com emicizumabe (fator VIII) em dose de 90 mg por semana.

O exame físico mostrou aumento de volume, com circunferência da coxa direita de 56 cm (22 cm maior que a coxa esquerda). A palpação revelou dor e endurecimento; os pulsos periféricos, poplíteos, pediosos dorsais e tibiais posteriores estavam presentes. Não foram observados distúrbios sensoriais ou motores ( Fig. 1 ).

**Fig. 1:** Exame físico mostrando que a circunferência da coxa direita era maior que a do membro contralateral. **Fonte:** *Arquivo dos autores.*

À admissão, o paciente apresentava níveis de hemoglobina (HB) ligeiramente diminuídos e contagem de plaquetas de 184.000 células/uL. Estes níveis continuaram dentro dos limites normais durante toda a hospitalização.

## Imagens Radiológicas

O paciente foi submetido a um ultrassom de tecido mole anterior que mostrou três coleções predominantemente líquidas no terço médio e na parte interna da coxa que se estendiam em direção aos planos musculares. A maior delas media 142*120*132 mm e tinha um volume de 1000 mL. As outras duas coleções adjacentes tinham volumes de 127 mL e 90 mL, respectivamente.

Uma angiotomografia computadorizada foi solicitada para avaliação da integridade vascular e demonstrou coleção loculada medindo 86*86*228 mm com volume estimado de 884 mL no plano muscular na região posteromedial da coxa direita e características sugerindo componente hemático deslocando anteriormente o feixe vascular do membro ( Figs. 2 e 3 ).

**Fig. 2:** Coleção loculada no plano muscular na região posteromedial da coxa direita. A diferença entre as circunferências das coxas direita e contralateral é óbvia. ( **A** ) Angiotomografia computadorizada em vista axial mostrando o pinçamento do feixe vascular. ( **B** ) A angiotomografia computadorizada em vista coronal revela o tecido capsular ao redor da lesão. **Fonte:** *Arquivo dos autores.*

**Fig. 3:** ( **A** ) Corte axial de ressonância magnética ponderada em T1 mostrando a presença de uma coleção medindo 103*57*182 mm de diâmetro no compartimento medial da coxa direita. ( **B** ) Corte coronal de ressonância magnética ponderada em T1 mostrando múltiplas coleções septadas (indicadas pela seta branca) estendendo-se ao tecido celular subcutâneo. **Fonte:** *Arquivo dos autores.*

## Tratamento

O paciente foi submetido à excisão cirúrgica. Durante a indução anestésica, recebeu uma primeira dose de 90 mcg/kg de fator VIIa recombinante (rFVIIa) (NovoSeven) por via intravenosa (IV) e, em seguida, 50 mcg/kg por 21 dias após a intervenção. A abordagem foi retrovastolateral direta e os achados intraoperatórios mostraram uma massa hemorrágica encapsulada e bem definida que fazia contato e deslocava o feixe vascular do membro. O tecido capsular e 2.000 mL de conteúdo hemático foram excisados. A drenagem percutânea foi mantida por três dias. Um segundo procedimento cirúrgico foi realizado devido a um aumento de diâmetro, com remoção de 400 mL de conteúdo hemático. O paciente continuou a terapia sistêmica por 21 dias e recebeu alta hospitalar sem complicações.

O acompanhamento do paciente após a alta hospitalar foi complexo. Devido às leis de isolamento da COVID-19, o controle presencial era inviável. Assim, foram realizados dois acompanhamentos telefônicos. No primeiro telefonema, realizado três meses após a alta hospitalar, o paciente referiu evolução clínica adequada, negando novos episódios de sangramento. Doze meses depois, no segundo telefonema de acompanhamento, ele referiu novo episódio de sangramento com necessidade de aplicação de fator VIIa recombinante.

## Discussão

A base fisiopatológica dos pseudotumores hemofílicos é o sangramento recorrente que causa hematomas encapsulados e aparência tumoral. Estes pseudotumores podem erodir as estruturas ósseas adjacentes. 2 3 A maioria dos casos apresenta histórico de trauma ou sangramento repetido devido à deficiência do fator VIII.

Clinicamente, os pseudotumores hemofílicos se apresentam como uma massa expansiva e indolor que se infiltra na maior parte do membro acometido. Estas neoplasias podem ser observadas no esqueleto axial ou em tecidos não ósseos, como os pulmões ou órgãos intra-abdominais. Os pseudotumores hemofílicos no esqueleto apendicular podem estar associados à limitação do movimento articular e déficits vasculares ou neurológicos em casos raros.

Há poucos sistemas de classificação, como o de Gilbert (de acordo com a localização) e PUMCH, que considera a localização, a extensão e o comprometimento ósseo.

O tratamento inclui observação e drenagem percutânea, mas a maioria dos pseudotumores hemofílicos não responde à terapia conservativa. A excisão cirúrgica e a reposição sistêmica de fator são os tratamentos de escolha na maioria dos casos.

Um pseudotumor hemofílico incontrolável, grande ou progressivo que não seja tratado pode afetar a integridade do membro, incluindo compressão vascular, déficits neurológicos ou erosão óssea/fraturas patológicas.

O desenvolvimento relativamente recente do rFVIIa permitiu que pacientes com altos títulos de inibidores dos fatores VIII e IX passem por procedimentos cirúrgicos com segurança. O rFVIIa é um agente hemostático confiável que não tem efeitos colaterais tromboembólicos. No entanto, pode ocorrer sangramento recorrente apesar do tratamento apropriado. 1 2 8
